# Comparison of Rifaximin Monotherapy and Rifaximin Combined with Probiotics in Patients with Irritable Bowel Syndrome: A Randomized Controlled Trial

**DOI:** 10.3390/nu17050763

**Published:** 2025-02-21

**Authors:** Chang Kyo Oh, Hwe Hoon Chung, Yu Jin Kim, Jin Bae Kim

**Affiliations:** Division of Gastroenterology, Department of Internal Medicine, Kangnam Sacred Heart Hospital, College of Medicine, Hallym University of Korea, Seoul 07441, Republic of Korea; hwehoon@hallym.or.kr (H.H.C.); kimyj@hallym.or.kr (Y.J.K.); jbkim87@hallym.or.kr (J.B.K.)

**Keywords:** rifaximin, probiotics, irritable bowel syndrome, randomized controlled trial

## Abstract

**Background/Objective**: Rifaximin is a nonabsorbable antibiotic used to treat irritable bowel syndrome (IBS). Recent studies on *Helicobacter pylori* eradication treatment have reported synergistic effects and low adverse effects when antibiotics are used in combination with probiotics; yet, such studies have not been conducted in IBS. Probiotics can enhance gut microbiota modulation, inhibition of pathogen adhesion to the gut epithelia, improvement in gut barrier function, anti-inflammatory effects, and improvement of gut immunity. Therefore, this study aimed to investigate the efficacy and safety of rifaximin in combination with probiotics compared to rifaximin monotherapy in patients with IBS. **Methods**: Patients with IBS were randomly allocated to receive rifaximin monotherapy or a combination of rifaximin and probiotics. The primary outcome was the response rate of the total IBS severity scoring system (IBS-SSS) score (>50-point decrease). Secondary outcomes were based on the response rate of the IBS quality of life (IBS-QOL) score and the IBS-SSS_1_ subscore (>10-point decrease in both scores). **Results**: Among 70 patients, the responder rates for the total IBS-SSS score were 65.7% in the combination therapy group and 31.4% in the monotherapy group at weeks 4 and 8, respectively (*p* = 0.004). The responder rates for IBS-QOL were 65.7% versus (vs.) 37.1% and 65.7% vs. 34.2% at weeks 4 and 8, respectively (*p* = 0.017 and *p* = 0.009, respectively). The IBS-SSS_1_ subscore responder rates were 65.7% vs. 40.0% at week 4 and 68.6% vs. 37.1% at 8 weeks (*p* = 0.031 and *p* = 0.017, respectively). **Conclusions:** Rifaximin combined with probiotics was superior to rifaximin monotherapy in patients with IBS. This combination therapy is considered an effective and safe treatment option for patients with IBS. However, further studies are needed to investigate the mechanisms of therapy and long-term outcomes.

## 1. Introduction

Irritable bowel syndrome (IBS) is a relapsing brain–gut interaction disorder characterized by recurrent abdominal pain, bloating, or a change in bowel habits [1]. A study from the United States, the United Kingdom, and Canada using the ROME IV criteria reported a prevalence of 5–7% in the population [2]. IBS causes various symptoms that lower the patient’s quality of life (QOL) and significantly increase social healthcare costs [3].

The pathophysiology of IBS is not clearly established, although multifactorial mechanisms, including gut microbiota, gut mucosal inflammation, visceral hypersensitivity, altered intestinal permeability, and brain–gut interaction have been proposed [1,4,5]. The gut microbiota in patients with IBS is significantly altered compared with that of healthy populations [5]. Patients with IBS have dysbiosis and low diversity of microbiota, which is associated with increased symptom severity [6,7]. Given these results, the gut microbiota appears to be a key factor that may play an important role in the pathophysiology of IBS [8]. Therefore, therapeutic strategies targeting the gut microbiota, aimed at improving dysbiosis and limiting the colonization of pathogenic bacteria, have been proposed as potential treatments for IBS. Accordingly, several studies have been conducted on probiotics, prebiotics, and antibiotics, and microbiome modulation therapies [9,10,11,12].

Rifaximin, a nonabsorbable antibiotic, has been proven effective in IBS [13,14], as it can change the gut microbiota and exert anti-inflammatory effects [12]. This effect of rifaximin is similar to the effects of probiotics, which have been frequently studied recently. In several studies, rifaximin has shown an effect from 2 weeks and improvement in symptoms in patients with IBS faster than probiotics, which often show improvement in symptoms after 4 weeks or more [13,14,15,16,17].

Recent studies on *Helicobacter pylori* eradication treatment have reported synergistic effects and low adverse effects when antibiotics are used in combination with probiotics compared to using antibiotics alone [18,19]. However, there have been no studies on rifaximin in combination with probiotics in IBS. Our previous study confirmed the efficacy and safety of probiotics in IBS [20]. Therefore, this study aimed to compare the efficacy and safety of rifaximin monotherapy and rifaximin in combination with probiotic therapy in patients with IBS.

## 2. Materials and Methods

### 2.1. Study Design

This prospective, randomized, open-label, controlled trial was conducted at Kangnam Sacred Heart Hospital, Hallym University of Korea, Seoul, Republic of Korea. Patients with IBS were randomly allocated to the rifaximin monotherapy group (monotherapy group) or rifaximin in combination with probiotics group (combination therapy group).

This study was conducted in accordance with the Declaration of Helsinki, and the study protocol was approved by the local institutional review board (approval number: HKS202303003, approval date: 24 May 2023). All the patients provided written informed consent before participation. This study also followed the Consolidated Standards of Reporting Trials guidelines and was registered with the International Clinical Trials Registry Platform (identifier: KCT0008556).

### 2.2. Study Participants

The participants were prospectively enrolled between October 2023 and August 2024. The inclusion criteria included age > 19 years and a diagnosis of IBS according to the ROME IV criteria. Eligible patients had recurrent abdominal pain, occurring on average at least once per week over the past 3 months and associated with two or more of the following criteria: (1) abdominal pain related to defecation, (2) abdominal pain related to change in stool frequency, and (3) abdominal pain related to change in stool appearance. IBS symptoms must have been present in the last 3 months, with symptom onset at least 6 months prior to diagnosis. The exclusion criteria were as follows: (1) treatment with prebiotics, antibiotics, probiotics, microbiome therapies, or fecal microbiota transplantation (FMT) within 2 weeks of enrollment; (2) a history of abdominal surgery (excluding appendectomy or cesarean section); (3) a history of inflammatory bowel disease, major psychiatric disease, or depression requiring medication; and (4) severe cardiopulmonary disease or malignancy. Baseline characteristics, including demographic and IBS symptom-related data, were collected from all the enrolled participants.

### 2.3. Intervention

Participants were randomly allocated 1:1 to the monotherapy or combination therapy group. The monotherapy group consisted of open-label treatment with rifaximin (200 mg four times daily for 14 days), whereas the combination therapy group consisted of open-label treatment with rifaximin (200 mg four times daily for 14 days) and probiotics (once daily for 28 days). Participants consumed the assigned study probiotics product orally once daily before a meal, with a glass of water over the 4-week study period. The daily dose was 1 × 10^10^ colony-forming units (CFUs). The multi-strain probiotic contained six strains: *Lactobacillus gasseri*, *Lactobacillus rhamnosus*, *Bifidobacterium longum*, *Bifidobacterium breve*, *Bifidobacterium bifidum*, and *Bifidobacterium lactis* at a dose of 1 × 10^10^ CFUs.

### 2.4. Questionnaires

#### 2.4.1. IBS Severity Scoring System (IBS-SSS)

The IBS-SSS comprises five subscore visual analog scales that assess abdominal pain severity (IBS-SSS_1_), abdominal pain frequency (IBS-SSS_2_), abdominal discomfort severity (IBS-SSS_3_), dissatisfaction with bowel habits (IBS-SSS_4_), and life interference in general (IBS-SSS_5_) [21], with total scores ranging from 0 to 500. Patients were classified as follows: <75 indicates remission; 75–175, mild disease; 175–300, moderate disease; and ≥300, severe disease.

#### 2.4.2. IBS-QOL

The IBS-QOL evaluates eight health subscales, each rated on a 5-point scale with total scores ranging from 0 to 100 [22].

### 2.5. Outcome Parameters

The primary outcome was the proportion of responders who experienced a >50-point decrease in the total IBS-SSS score, which ranges from 0 to 500. The mean values for individual symptoms and total IBS-SSS scores were calculated at baseline and weeks 2, 4, and 8. Secondary outcomes included the proportion of responders with a reduction of >10 points in the IBS-SSS_1_ subscore and IBS-QOL scores. These scores were calculated at baseline and weeks 2, 4, and 8.

All patients were scheduled to visit our outpatient clinic at weeks 2, 4, and 8 to monitor adverse events following treatment with rifaximin monotherapy or rifaximin in combination with probiotics. During these visits, the participants were also asked about the occurrence of abdominal pain, headache, diarrhea, nausea, bloating, vomiting, and other adverse events.

### 2.6. Sample Size Calculation

On the basis of previous studies, we estimated the rifaximin response rate to be up to 30–40% [13,14,23,24]. In our preliminary experience with rifaximin and probiotics in patients with IBS, we presumed responder rates of 66% in the combination therapy group and 33% in the monotherapy group. Thus, a sample size of 33 patients per group was required to demonstrate superiority, assuming an alpha level of 5% and power of 80%. Therefore, the required sample size was 70 patients, accounting for a dropout rate of 5%.

### 2.7. Randomization

A block randomization method (block sizes of two and four) was applied. A research assistant who was not involved in clinical practice generated the random allocation sequence, and the contents were concealed until the intervention group was assigned at the time of treatment.

### 2.8. Statistical Analysis

The primary outcome was analyzed using intention-to-treat analysis, which included all patients who underwent randomization. Continuous outcomes were analyzed and compared using the paired *t*-test. Categorical outcomes were compared using the chi-square test or Fisher exact test. Statistical significance was set at *p* < 0.05 (two-sided), and all statistical analyses were performed using the SPSS statistical software (version 21.0; IBM Corp., Armonk, NY, USA).

## 3. Results

### 3.1. Patient Characteristics

**Figure 1:**
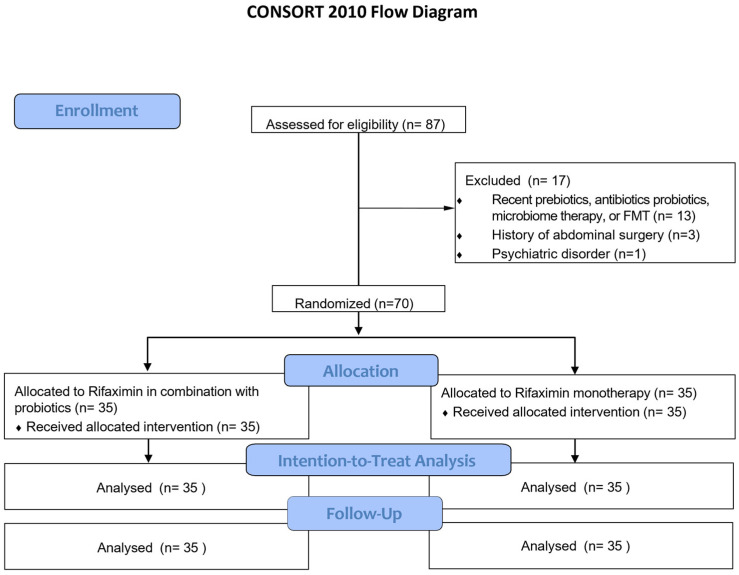
The flow diagram of this study. FMT, fecal microbiota transplantation.

A total of 87 patients were enrolled between October 2023 and August 2024. Among them, 17 patients were excluded because they were recently treated with prebiotics, antibiotics, probiotics, microbiome therapies, or FMT (*n* = 13); had a history of abdominal surgery (*n* = 3); or had a psychiatric disorder (*n* = 1). Finally, 70 patients were randomly allocated to receive either monotherapy or combination therapy. No patients were lost to follow-up to confirm outcomes or adverse events (Figure 1).

Table 1 shows the baseline characteristics of the patients in the two groups. The mean ages (standard deviation) were 59 (17.4) and 59 (15.9) years in the monotherapy and combination therapy groups, respectively, and there were 42.9% and 37.1% males, respectively. A total of 68.6% and 60.0% of patients in the monotherapy and combination therapy groups, respectively, received at least one prior IBS treatment that was not expected to interfere with the study treatment. According to the ROME IV criteria, most patients in the monotherapy and combination therapy groups were diagnosed with IBS-D (74.3% and 77.1%, respectively), followed by IBS-M (25.7% and 20.0%, respectively) and IBS-C subtypes (0% and 2.9%, respectively). At baseline, the mean scores for the total IBS-SSS and IBS-QOL were 335.8 and 325.9 and 65.7 and 63.6 in the monotherapy and combination therapy groups, respectively. Among these patients, 60.0% and 57.1% had severe disease and 40.0% and 42.9% had moderate disease, respectively. No patient had previously received FMT or probiotics within the 4 weeks between the two groups.

### 3.2. Outcomes

**Figure 2:**
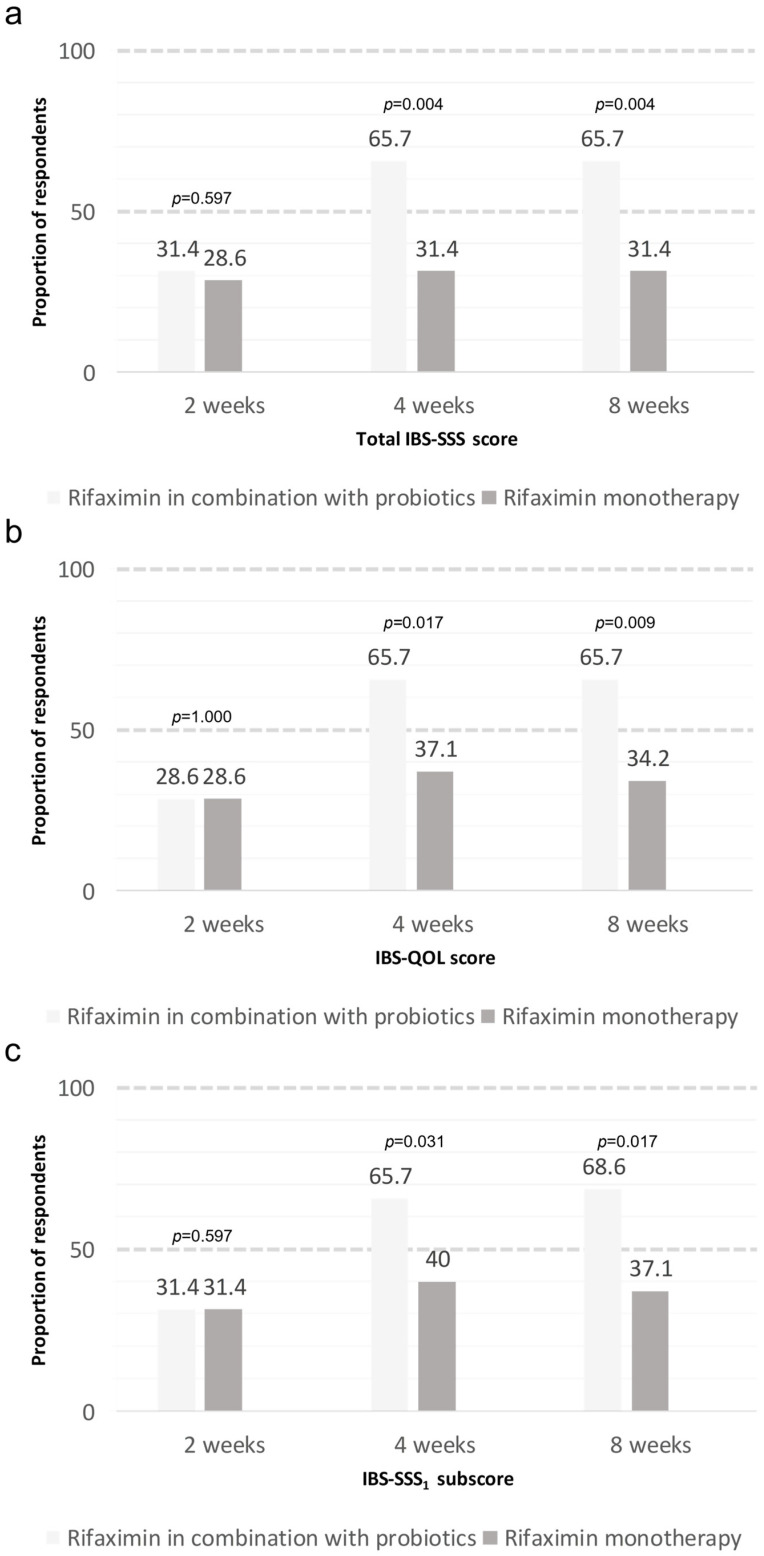
The proportion of patients reported as responders for primary and secondary outcomes: (**a**) total IBS-SSS score; (**b**) IBS-QOL score; and (**c**) IBS-SSS1 score (abdominal pain severity).

At week 4, the response rates of the combination therapy and monotherapy groups for the total IBS-SSS score were 65.7% and 31.4%, respectively (*p* = 0.004). The response rates for the total IBS-SSS score were 65.7% in the combination therapy group and 31.4% in the monotherapy group at week 8 (*p* = 0.004). Responders maintained their responses from week 4 to week 8. The response rates were 31.4% and 28.6% in the combination therapy and monotherapy groups, respectively, for the total IBS-SSS score at week 2 (*p* = 0.597) (Figure 2).

IBS-QOL responder rates in the combination therapy and monotherapy groups were 28.6% versus (vs.) 28.6%, 65.7% vs. 37.1%, and 65.7% vs. 34.2% at weeks 2, 4, and 8, respectively (*p* = 1.000, *p* = 0.017, and *p* = 0.009, respectively). IBS-SSS_1_ subscore responder rates in the combination therapy and monotherapy groups were 31.4% vs. 31.4%, 65.7% vs. 40.0%, and 68.6% vs. 37.1% at weeks 2, 4, and 8, respectively (*p* = 1.000, *p* = 0.031, and *p* = 0.017, respectively) (Figure 2).

The change in total IBS-SSS score significantly improved after weeks 4 and 8 in the combination therapy group compared with the monotherapy group (−27.1 ± 32.8 vs. −101.8 ± 76.9, respectively; *p* < 0.001). The change in the total IBS-SSS score at week 2 was similar between the groups (Table 2). Furthermore, all IBS-SSS subscores and IBS-QOL scores in the combination therapy group significantly improved after weeks 4 and 8 compared with those in the monotherapy group (Table 2). The changes in the IBS-SSS subscores and IBS-QOL scores at week 2 were similar in both groups.

No severe adverse effects were observed. At the beginning of this study, adverse events occurred in three participants (8.6%) in the combination therapy group and six participants (17.1%) in the monotherapy group within 2 weeks (*p* = 0.477). The reported symptoms resolved within 4 weeks (Table 3). In the combination therapy group, diarrhea, nausea, and bloating occurred in one participant (2.9%). In the monotherapy group, headache occurred in one participant (2.9%), diarrhea in two participants (5.7%), nausea in two participants (5.7%), and bloating in one participant (2.9%).

## 4. Discussion

This study is the first to show that rifaximin in combination with probiotics is effective and safe for patients diagnosed with ROME IV IBS. Regarding the total IBS-SSS score, rifaximin in combination with probiotics did not show a significant difference from the rifaximin monotherapy group at week 2. However, there was a significant difference in improvement from weeks 4 to 8.

The combination therapy group showed response rates of approximately 30% at week 2 and 60% at weeks 4 and 8. This is a high response rate compared with that reported in previous studies conducted with rifaximin. However, the results at weeks 4 and 8 were similar to those of previous studies on probiotics. The IBS-SSS and IBS-QOL scores showed that rifaximin in combination with probiotics significantly improved abdominal pain severity and quality of life. Similar to the total IBS-SSS score, the IBS-SSS₁ subscore and IBS-QOL scores were similar at week 2, but showed significant differences from week 4 to week 8.

Rifaximin was used in the treatment of IBS, and its efficacy was reported in TARGET 1,2,3, a randomized controlled trial targeting non-IBS-C patients [13,14]. Additionally, the efficacy of rifaximin was proven in another trial targeting IBS-C [23,24]. Although the efficacy of rifaximin has been clearly proven in several studies, the mechanism responsible for this has not been clearly established. Nevertheless, several studies have reported that rifaximin affects the gut microbiota of patients with IBS and may help improve symptoms [12,25].

The gut microbiota affects human health and disease, and the fact that approximately 10% of patients with IBS have post-infectious IBS has led to many studies on the relationship between IBS and the gut microbiota [12,25]. The abundances of *Lactobacillus* and *Bifidobacterium* were observed to be lower in patients with IBS than in healthy controls. Several studies have reported that half to two-thirds of patients had dysbiosis. Moreover, Vervier et al. reported that pathogen-like microbiota were identified in half of the IBS group, and dysbiosis was restored and symptoms improved in this group through a low-fermentable oligosaccharide, disaccharide, monosaccharide, and polyol diet [26]. In our previous study, approximately 60% of patients with IBS had improved symptoms in the probiotic group [20]. Hence, it seems that microbiome modulation therapy is effective in IBS patients with dysbiosis. In our study, a detailed analysis of responders to rifaximin and probiotics was difficult because microbiome analysis was not performed. However, considering that those who responded to rifaximin also responded to probiotics at week 4, we speculate that rifaximin and probiotics helped improve symptoms through similar mechanisms.

Our study showed that treatment duration varies depending on the treatment type, and appropriate treatment duration seems to play an important role. Previous studies have reported that rifaximin improves symptoms from week 2 [13,14]. In contrast, previous studies conducted on probiotics have reported that symptoms improve starting from week 4 or 8 of treatment [15,16,17,20]. In our study, similar responder rates were reported in the two groups administered rifaximin at 2 weeks, and the combination therapy group showed a significantly higher response rate than the monotherapy group at 4 and 8 weeks. In contrast to our previous results with probiotic monotherapy, both groups treated with rifaximin showed higher symptom improvement in the second week. Additionally, the effect of rifaximin was confirmed to last up to week 8 of rifaximin monotherapy. Thus, it seems that administering a combination of rifaximin for 2 weeks and probiotics for 4 weeks can be helpful in improving symptoms in patients with IBS.

Rifaximin can cause adverse effects, and previous studies on rifaximin in IBS reported that adverse effects occurred in 10–18% of patients [27,28]. Our study did not show a significant difference, but the combination therapy group showed fewer side effects than the monotherapy group. In a study on the eradication of *Helicobacter pylori*, where many studies on combination therapy with antibiotics and probiotics were conducted, combination therapy had the effect of reducing adverse effects compared with antibiotic monotherapy [18,19]. Regarding adverse effects, it seems necessary to confirm the difference between monotherapy and combination therapy in further large-scale studies.

This study has several limitations. First, this was an open-label, randomized controlled trial, and not a placebo-controlled study. Second, this study only collected short-term data at 2, 4, and 8 weeks, so long-term data are lacking. Third, although all subtypes of IBS were included, the proportion of patients with IBS-M and IBS-C was small; therefore, it may be difficult to say whether this study had sufficient power. Lastly, because gut microbiota analysis was not performed, it was difficult to determine whether gut microbiota changes were accompanied by or related to the improvement of patients’ symptoms.

Although many randomized controlled trials have been performed using probiotics for the treatment of IBS, an overall assessment of their efficacy remains inconclusive. The beneficial effects of probiotics in IBS may vary depending on the probiotic strains and the heterogeneity of the patient population with IBS. Identifying and validating the microbiota that correlates with those who respond to rifaximin in combination with probiotics is important because it may allow for better stratification and selection of patients who are likely to benefit from combination therapy. Future studies are needed to identify biomarkers that can predict patient subgroups responding to rifaximin in combination with probiotics.

## 5. Conclusions

Rifaximin in combination with probiotics was effective and safe in improving IBS severity and patient QOL. However, further studies are needed to accurately identify the mechanism of symptom improvement and long-term outcomes according to the combination therapy of rifaximin and probiotics.

## Figures and Tables

**Table 1:** Baseline characteristics of the patients.

Characteristics	Combination Therapy Group (*n* = 35)	Monotherapy Group (*n* = 35)	*p*
Age, mean (SD), years	59 (17.4)	59 (15.9)	0.505
Sex, male, *n* (%)	15 (42.9)	13 (37.1)	0.626
BMI, mean (SD), kg/m^2^	24.1	23.9	0.971
BMI class, *n* (%)			0.653
Normal	21 (60.0)	24 (68.6)	
Underweight	2 (5.7)	2 (5.7)	
Overweight	8 (22.9)	4 (11.4)	
Obese	4 (11.4)	5 (14.3)	
Type of IBS, *n* (%)			0.777
IBS-D	26 (74.3)	27 (77.1)	
IBS-M	9 (25.7)	7 (20.0)	
IBS-C	0	1 (2.9)	
IBS-SSS class, *n* (%)			0.808
Moderate	14 (40.0)	15 (42.9)	
Severe	21 (60.0)	20 (57.1)	
IBS-SSS score, mean, (SD)			
Total	335.8 (24.6)	325.9 (31.9)	0.150
Abdominal pain severity (IBS-SSS_1_)	72.5 (4.2)	70.9 (5.5)	0.192
Abdominal pain frequency (IBS-SSS_2_)	63.1 (7.2)	61.4 (8.8)	0.375
Abdominal discomfort severity (IBS-SSS_3_)	69.1 (5.6)	67.2 (6.7)	0.197
Bowel habit dissatisfaction (IBS-SSS_4_)	64.4 (6.1)	61.5 (8.2)	0.101
Life interference in general (IBS-SSS_5_)	66.7 (6.5)	64.8 (8.6)	0.315
IBS-QOL score, mean, (SD)	65.7 (5.7)	63.6 (6.7)	0.161
Previous medication use, *n* (%)	24 (68.6)	21 (60.0)	0.454
Previous FMT or probiotics use within 4 weeks, *n* (%)	0	0	

**Table 2:** Change in irritable bowel syndrome severity scoring system score and irritable bowel syndrome quality of life score from baseline to 2 weeks and 4 weeks.

	Combination Therapy Group (*n* = 35)	Monotherapy Group (*n* = 35)	*p*
**2 weeks**			
IBS-SSS score, mean, (SD)			
Total	−36.7 (32.3)	−28.5 (35.6)	0.316
Abdominal pain severity (IBS-SSS_1_)	−8.8 (6.3)	−7.0 (7.4)	0.302
Abdominal pain frequency (IBS-SSS_2_)	−6.3 (7.7)	−4.8 (7.7)	0.444
Abdominal discomfort severity (IBS-SSS_3_)	−8.5 (7.3)	−7.2 (8.1)	0.508
Bowel habit dissatisfaction (IBS-SSS_4_)	−6.7 (7.9)	−4.7 (8.6)	0.308
Life interference in general (IBS-SSS_5_)	−6.5 (6.8)	−5.3 (8.1)	0.516
IBS-QOL score, mean, (SD)	−7.0 (6.5)	−5.6 (6.9)	0.376
**4 weeks**			
IBS-SSS score, mean, (SD)			
Total	−109.7 (72.0)	−65.7 (73.7)	0.014
Abdominal pain severity (IBS-SSS_1_)	−25.4 (17.4)	−15.4 (17.0)	0.018
Abdominal pain frequency (IBS-SSS_2_)	−21.1 (13.7)	−12.6 (14.7)	0.014
Abdominal discomfort severity (IBS-SSS_3_)	−21.4 (13.9)	−14.3 (14.3)	0.039
Bowel habit dissatisfaction (IBS-SSS_4_)	−19.0 (14.1)-	−9.9 (14.7)	0.011
Life interference in general (IBS-SSS_5_)	−21.4 (16.0)	−13.5 (16.6)	0.046
IBS-QOL score, mean, (SD)	−20.3 (16.1)	−12.5 (15.9)	0.045
**8 weeks**			
IBS-SSS score, mean, (SD)			
Total	−109.9 (61.8)	−67.6 (65.2)	0.007
Abdominal pain severity (IBS-SSS_1_)	−24.3 (14.9)	−15.9 (14.4)	0.018
Abdominal pain frequency (IBS-SSS_2_)	−20.9 (12.2)	−12.1 (13.2)	0.006
Abdominal discomfort severity (IBS-SSS_3_)	−21.9 (13.0)	−15.1 (13.3)	0.033
Bowel habit dissatisfaction (IBS-SSS_4_)	−20.8 (11.6)	−10.2 (13.8)	0.001
Life interference in general (IBS-SSS_5_)	−21.7 (15.8)	−14.2 (16.0)	0.055
IBS-QOL score, mean, (SD)	−20.0 (14.8)	−14.4 (13.7)	0.103

**Table 3:** Incidence of adverse events during study periods.

	Combination Therapy Group (*n* = 35)	Monotherapy Group (*n* = 35)	*p*
Total, *n* (%)	3 (8.6)	6 (17.1)	0.477
Abdominal pain, *n* (%)	0	0	
Headache, *n* (%)	0	1 (2.9)	1.000
Diarrhea, *n* (%)	1 (2.9)	2 (5.7)	1.000
Nausea, *n* (%)	1 (2.9)	2 (5.7)	1.000
Bloating, *n* (%)	1 (2.9)	1 (2.9)	1.000
Vomiting, *n* (%)	0	0	

## Data Availability

The data in this study are not publicly available but can be requested from the corresponding author.

## Abbreviations

The following abbreviations are used in this manuscript:

IBS	irritable bowel syndrome
QOL	quality of life
FMT	fecal microbiota transplantation
CFUs	colony-forming units
IBS-SSS	irritable bowel syndrome severity scoring system
